# Disease-modifying factors in hereditary angioedema: an RNA expression-based screening

**DOI:** 10.1186/1750-1172-8-77

**Published:** 2013-05-20

**Authors:** Alberto López-Lera, Fátima Sánchez Cabo, Sofía Garrido, Ana Dopazo, Margarita López-Trascasa

**Affiliations:** 1 Immunology Unit, Hospital Universitario La Paz and Hospital La Paz Research Institute (IdiPAZ), Madrid, Spain; 2 Centre for Biomedical Network Research on Rare Diseases (CIBERER), Instituto de Salud Carlos III (ISCIII), Madrid, Spain; 3 Genomics Unit, Centro Nacional de Investigaciones Cardiovasculares, (CNIC), Madrid, Spain

**Keywords:** Disease-modifying factor, Expression microarray, Hereditary angioedema, Infection

## Abstract

**Background:**

Hereditary Angioedema due to C1-Inhibitor deficiency (HAE types I and II) is a monogenic disease characterized by sudden, self-limited episodes of cutaneous and mucosal swelling due to local deregulation of vascular permeability. Despite its monogenic pattern of inheritance, HAE exhibits great clinical variability and low genotype/phenotype correlation among those affected, which ultimately hinders therapeutic approach and probably underlies yet unknown genetic and environmental factors.

**Methods:**

We studied whole-genome RNA expression of PBMCs in three HAE type-I families (accounting for 40 individuals), 24 of which carry the same R472X mutation in the C1-Inhibitor gene and show large variability in terms of disease expression. Those included in this study were analyzed according to the presence of mutation and/or clinical symptoms.

**Results:**

Instead of a single, common disease-associated expression pattern, we found different transcriptome signatures in two of the families studied. In one of them (referred to as DR family), symptoms correlate with the upregulation of 35 genes associated to the biological response to viral infections (including RSADs, OAS, MX and ISG pathway members) and immune response. In another pedigree (Q family), disease manifestation is linked to the upregulation of 43 genes with diverse functions, including transcription and protein folding. Moreover, symptoms-free members of the Q pedigree display relatively higher expression of 394 genes with a wide diversity of functions.

**Conclusion:**

We found no evidence for a common altered PBMC expression pattern linked to HAE symptoms in the three families analyzed. All the data considered, differential gene expression in PBMCs do not seem to play a significant role in the predisposition or protection against HAE in the basal -between crises- conditions analyzed. Although the RNA expression pattern associated to the response to viral infections observed in the DR family supports the idea of infectious diseases as a modifying factor for HAE severity, large-scale studies would be needed to statistically associate such expression pattern to the development of this rare disease.

## Introduction

Hereditary Angioedema (HAE, OMIM#106100) is a rare genetic condition characterised by the occurrence of severely incapacitating episodes of edema affecting mucosal and submucosal layers. Edema attacks in HAE develop spontaneously in any body location (face, limbs, genitals or upper respiratory and gastrointestinal tracts) and can lead to life-threatening suffocation when there’s involvement of the upper airways [1].

Inheritance of the disease is autosomal dominant due to deleterious mutations in the serpin (Serum protease inhibitor) C1-Inhibitor (C1-INH) (HAE types I and II) or gain-of-function mutations in the gene coding for the coagulation Factor XII (FXII) (HAE type III or oestrogen-related HAE) [2]. Both genetic alterations ultimately cause uncontrolled activation of the contact and kinin-forming cascades, as evidenced by the extensive activation of the factor XII–dependent pathways and bradykinin release observed in HAE patients’ plasmas [3,4]. Bradykinin, through its interaction with its specific receptor on the endothelium surface, is considered the major mediator of edema in these patients [5].

Systematic genetic screening has been performed in HAE accounting for more than 200 mutations described to date in the *C1INH* locus (responsible for the majority of HAE cases). However, little or no genotype-phenotype correlation has been detected [6]. Patients with HAE carrying the same mutation exhibit large variability of symptoms’ frequency and severity, even among siblings. Such variability hinders therapeutic assessment and probably underlies yet unknown genetic and environmental factors.

A potential source of clinical variation comes from the poorly studied role of adaptive immunity in HAE. Although some reports exist indicating abnormal distribution of T lymphocyte surface receptors, reduced Langerhans cell numbers and abnormal T- and B-cell counts in HAE patients [7,8], little is known about how adaptive immunity function and variation may modify the clinical expression of the disease. More precisely, how innate and adaptive immunity crosstalk in dealing with infection is of particular interest, as viral and bacterial infections are known to induce hives and edema under certain pathological situations.

In the present report, we intended to study the contribution of peripheral blood mononuclear cells (PBMCs) to the clinical expression of HAE. For this purpose, the RNA expression profile of PBMCs from 3 large HAE type I families was analyzed according to the presence of mutation in the *C1INH* locus (R472X) and the existence or frequency of clinical symptoms of angioedema. Our results did not evidence common alterations in the expression pattern of PBMCs in association to the frequency and severity of disease manifestations in the 3 families. Instead, different expression profiles associated to disease manifestation in each family, suggesting that the PBMC compartment does not make a significant contribution to HAE pathology in basal, non-inflammatory conditions.

## Materials and methods

### Ethics statement

Biological sampling of human PBMCs was carried out upon informed consent from the participants. The ethical committee of Hospital La Paz gave approval to these studies.

### Patients and samples

HAE families were selected for these experiments on the basis of two criteria: (i) the presence of a common mutation in the *C1INH* locus segregating in the family and (ii) the existence of a wide variability of symptoms’ frequency among the affected individuals. Three non related families carrying the R472X mutation were chosen: family AR (16 members), family DR (9 members) and family Q (15 members) (Table 1). In each family, the index case was assigned number 0 (e.g. AR0), while the remaining family members received correlative numbers depending on their respective consanguinity (eg. AR1, AR2…). Additionally, those family members not carrying the R472X mutation were included in the study as healthy controls.

**Table 1:** Cohort descrip0074ion

**Family code**	**Gender**	**Age**	**Arg472Stop**	**[C1-INH] (mg/dL)**	**C1-INH activity (% of NHP)**	**Symptoms**	**Treatment needed**	**Severity score**	**Severity degree**
**Family AR**									
ARO	F	37	Yes	8.80	31.98	Yes	Androgens	43	Severe
AR2	F	64	Yes	6.84	25.11	Yes	Androgens	48	Severe
AR3	M	26	Yes	4.53	13.36	Yes	Androgens	48	Severe
AR4	M	60	Yes	7.51	25.06	Yes	Androgens	48	Severe
AR5	F	27	Yes	<2.98	21.46	Yes	No	24	Moderate
AR7	F	60	Yes	5.81	18.21	Yes	Androgens	43	Severe
AR8	F	26	Yes	3.56	10.84	Yes	Androgens	49	Severe
AR9	F	40	Yes	3.79	18.28	Yes	No	12	Mild
AR10	M	53	Yes	7.05	34.24	No	No	0	Asymptomatic
AR11	M	30	No	22.30	>100	-	-	-	-
AR12	F	56	No	19.90	>100	-	-	-	-
AR13	F	44	No	25.30	>100	-	-	-	-
AR14	M	51	No	23.90	>100	-	-	-	-
AR15	M	52	No	18.70	>100	-	-	-	-
AR16	M	45	No	25.80	>100	-	-	-	-
AR17	M	47	No	25.30	>100	-	-	-	-
**Family DR**									
DR0	F	52	Yes	3.65	24.8	Yes	No	17	Mild
DR1	M	81	Yes	5.72	22.41	No	ACE-i	0	Asymptomatic
DR3	M	32	Yes	5.07	26.70	No	No	0	Asymptomatic
DR4	M	26	Yes	5.49	20.47	No	No	0	Asymptomatic
DR5	F	17	No	24.30	>100	-	-	-	-
DR6	M	75	No	21.50	>100	-	-	-	-
DR7	M	41	No	24.90	>100	-	-	-	-
DR8	F	39	No	18.00	>100	-	-	-	-
DR9	F	37	No	20.30	>100	-	-	-	-
**Family Q**									
Q0	M	82	Yes	4.11	10.68	Yes	Androgens	48	Severe
Q3	F	86	Yes	7.61	25.44	No	No	0	Asymptomatic
Q4	F	57	Yes	11.80	40.63	Yes	Androgens	26	Moderate
Q6	F	36	Yes	4.64	16.42	Yes	Androgens	49	Severe
Q7	F	50	Yes	<2.98	9.76	Yes	No	25	Moderate
Q8	F	48	Yes	5.88	16.26	Yes	No	28	Moderate
Q9	F	24	Yes	3.27	14.92	Yes	No	48	Severe
Q10	F	71	Yes	6.28	17.23	No	-	0	Asymptomatic
Q11	M	56	No	21.30	>100	-	-	-	-
Q12	F	44	No	16.05	86.99	-	-	-	-
Q13	M	38	No	26.40	>100	-	-	-	-
Q14	F	47	No	20.30	>100	-	-	-	-
Q15	F	75	Yes	6.34	18.00	Yes	Androgens	25	Moderate
Q16	F	40	Yes	4.78	26.70	Yes	Androgens	27	Moderate
Q17	M	45	Yes			Yes	Androgens	25	Moderate

Three HAE families were included in the microarray study: AR (16 members), DR (9 members) and Q (15 members). Treatment received by the patient and the associated severity score at the time of sampling are shown. F: female; M: male; NHP: Normal Human Plasma; ACE-i: Angiotensin Converting Enzyme- inhibitors; *: Not determined.

For the statistical analyses, patients were clustered in clinical categories according to their disease severity index, as previously described [6] (Table 1).

In the DR family and based on the available experimental data dealing with the extent of androgen effects on C1-INH synthesis [9], treatment was suspended two weeks before blood sampling upon consent from their physicians. As demonstrated by Gelfand and colleagues, the effect of androgen treatment, as measured by the increase in C1-INH and C4 serum levels, is maximal by one to two weeks after the onset of the treatment but decreases rapidly once it is stopped. For this reason, we set two weeks after treatment removal as a convenient lapse of time for sample collection. None of the patients referred angioedema symptoms or other pathological conditions at the time of sampling.

For validation purposes and in order to investigate the permanence of the detected RNA expression patterns, duplicated samples (first extraction at the time of the microarray experiment and a second one, 10 months later) were extracted and handled as recommended by the manufacturer prior to processing. 2.5 mL of whole blood were collected into each PAXgene™ Blood RNA Tube (PreAnalytix® GmbH, Switzerland) using standard methods. RNA was isolated from blood samples using the PAXgene™ Blood RNA Kit (PreAnalytix). The quality and integrity of RNA were assessed both by agarose gel electrophoresis and bioanalyzer methods. Absorbances at 260, 230 and 280 nm were measured and the A260/280 and A260/230 ratios were considered adequate. Additionally, EDTA-plasma samples were separated and stored at -80°C until used.

### Labeling of RNA and microarray hybridization

One-Color Microarray-Based Gene Expression Analysis Protocol (Agilent Technologies®, Palo Alto, CA, USA) was used to amplify and label RNA. Briefly, 800 ng of total RNA from patients and controls, were reverse transcribed using T7 promoter primer and the Moloney murine leukemia virus (MMLV) reverse transcriptase (RT). cDNA was then converted to anti-sense RNA (aRNA) by using T7 RNA polymerase which amplifies target material simultaneously incorporating cyanine 3 (Cy3)-labeled CTP. Samples were hybridized to a Whole Human Genome Microarray™ 4x44K (G4845A, Agilent Technologies®). 1.65 micrograms of Cy3-labeled aRNA were hybridized for 17 hours at 65°C in an Agilent hybridization oven (G2545A, Agilent Technologies) set to 10 rpm in a final concentration of 1x GEx Hybridization Buffer HI-RPM (Agilent Technologies). Arrays were washed and dried out according to manufacturer’s instructions (One-Color Microarray-Based Gene Expression Analysis, Agilent Technologies), and scanned at 5 mm resolution on an Agilent DNA Microarray Scanner™ (G2565BA, Agilent Technologies®) using the default settings for 4x44k format one-color arrays. Images provided by the scanner were analyzed using Feature Extraction software v10.1.1.1™ (Agilent Technologies®).

### Preprocessing and data analysis

Raw signals were thresholded to 1 and quantiles normalization [10] was performed using GeneSpring™ software. Data were considered in the log2 scale. Default flags were considered as absent, except saturated spots that were flagged as marginal.

From the initial 34183 probes present in the chip, 20441 remained after applying the by-expression, by-flag and by-error data filtering. Based on statistical outliers criteria implemented in the Bioconductor package ArrayQualityMetrics™, four samples showing excessive variance among replicates (AR5, AR16, Q0 and Q4) were removed for further analysis. Two different statistical analyses were performed using the Limma™ package from Bioconductor: (i) patients with mutation *versus* patients without mutation, using the families as controlling variable (random factor) and (ii) symptomatic *versus* asymptomatic patients carrying mutation in each family.

### Analysis of microarray results

The web-based pathways analysis tool IPA (Ingenuity Systems®, http://www.ingenuity.com) was used to identify biological pathways affecting the development of edema in HAE. Gene expression was considered as significantly upregulated when Fold Change (FC) was above 2. The differentially expressed genes among symptomatic and asymptomatic HAE patients were classified with gene ontology (GO) analysis using the Database for Annotation, Visualization and Integrated Discovery (DAVID) v6.7. In this process, Fisher exact p values are used to measure the gene-enrichment in annotation terms while Benjamini scores provide an estimation of the False Discovery Rate (FDR). FDR is defined as the expected proportion of false positives among the declared significant results. Clustering results were considered significant only when both the p value and FDR statistics were below 0.05.

### Real time PCR

TaqMan custom assay primers and probes (Roche Diagnostics®, Barcelona, Spain) were designed by using the manufacturer’s Universal Probe Library Assay Design Center. In all cases, the RNA integrity and the specificity of RT-PCR amplified products were analyzed for by agarose-gel electrophoresis prior to quantification. Analysis of the results was performed with the standard curve method and using the GAPDH and β-actin genes for normalization. Equal amplification efficiencies were assessed for target and reference genes.

Total RNA (500 ng) from patients and controls was amplified using the SuperScript First Strand Synthesis System™ with random hexameres (Invitrogen®, Carlsbad, CA, USA). Real Time PCR was performed according to the manufacturer’s protocols in a LightCycler® Carousel-based System. All samples were loaded by duplicate and negative controls (no-template) were included in every experiment. RT-PCR experiments were conducted on RNA samples isolated from two independent blood extractions. The primers used for RT-qPCR validation were designed at Universal Probe Library Assay Design Centre (http://www.roche-applied-science.com/sis/rtpcr/upl/index.jsp?id=UP030000). The primers sequences were: RIG-I (NM_014314.3) 5′TGGACCCTACCTACATCCTGA3′ (F) and 5′GGCCCTTGTTGTTTTTCTCA3′ (R);IFIT1 (NM_001548.3) 5′AGAACGGCTGCCTAATTTACAG3′ (F) and 5′GCTCCAGACTATCCTTGACCTG3′ (R);ISG15 (NM_005101.3) 5′GCGAACTCATCTTTGCCAGT3′ (F) and 5′AGCATCTTCACCGTCAGGTC3′ (R);OAS3 (NM_006187.2).

### Protein analysis in serum

In order to study the cytokines signature of the patients’ sera, selected protein markers were quantified using an on-demand magnetic-bead-based multiplex immunoassay from Bio-Rad (Bio-Plex Cytokine Group I 10-plex Assay; Bio-Rad®, Hercules, CA, USA). The kit included beads, probes and reagents for the quantification of IL-2, IL-4, IL-5, IL-6, IL-8, IL-10, IL-12p70, IL-17a, IFN-γ and TNF-α in serum or plasma.

## Results

### Microarray results

For the evaluation of the results, a threshold of Fold Change (FC):2 was used in order to provide a conservative foundation for further interpretations. Overall analysis of the microarray outcome did not evidence alterations in the expression pattern of a single pathway in the HAE symptomatic individuals from our cohort. Instead, it suggests the involvement of different pathways in two of the families studied. Age and sex showed no major association to clinical frequency and severity in the series.

The experimental population was stratified in subgroups according to the “severity index” criteria and analyzed accordingly. Only those comparisons reaching significance are shown.

### Genes showing altered expression patterns in HAE symptomatic patients

Genes exhibiting altered patterns of expression in each family are enlisted in Table 2.

**Table 2:** Microarray results

**Family DR**	**Genes upregulated IH the symptomatic palielit DRO**			
	**Gene**	**FC**	**Adjpvalue**	**Description**
**Type I Interferon signaling pathway and /or Response to virus** **p value:7.3 × 10**^**-7 **^ **FDR: 1.2 × 10**^**-4**^	ISG15	11.87	0.0012	ISG15 ubiquitin-likemodifier(ISG15). [NM_005101]
	HERC5	11.63	0.0094	hect domain and RID 5 (HERC5). [NM_U 16323]
	OAS3	11.00	0,0094	2’-5’-oligoadenylate synthetase 3, l00kDa (OAS3) [NM_006187]
	IFI44	10.70	0,0318	interferon-induced protein 44 (IFI44). [NM_006417]
	1F1141.	17.03	0.0267	interferon-induced protein 44-like (IFI44L), mRNA [NM_006820]
	MXI	9.25	0,0241	myxovirus resistance 1 (MX1), [NM_002462]
	1F16	8.57	U.U1M	Interferon, alpha-inducible protein 6 (IFI6) variant 3 [NM_022873]
	IFIT3	861	00588	interfernn-indnced protein with tetratricnpeptide repeals 3 (IFIT3). [NM_00 1549]
	IFIT2	7.26	0,0096	interferon-induced protein with tetratricopeptide repeats 2 (IFIT2), [NM_00 1547]
	OASL	7.21	0,0485	2’-5’-oligoadenylate synthetase-like (OA.SL), variant 1, [NM 0037331]
	USP18	6.23	0,0080	ubquitin specific peptidase 18 (USP18), varient 1, [NM_017414]
	OAS2	5.89	0, 050	2’-5’-oligoadenylate synthetase 2 69/71/kDA (OAS2), variant 3, [NM_001032731]
	XAF1	4.96	0,0116	XIAP associated factor 1 (XAF1), variant 1, [NM 017523]
	RSAD2	29.85	0,0128	radical S-alenosyl methionine domain containing 2 (RSAD2 [NM_0S0657]
	IFIT1	20.25	0,0241	interferon-induced protein with tetratricopeptide repeats 1 (IFIT1), [NM 001548]
	IFIT35	2.71	0.050	interferon-induced protein 35 (IF135) [NM 005533]
**Endoplasmic reticulum unfolded protein response** **pvalue :3.8 × 10**^**-2**^ **FDR: 4 × 10**^**-2**^	HSP90AB1	7.84	1,35E-06	heat shock protein 9OkDa alpha, class B member 1 (HSP9OAB 1) [NM 007355]
	DERL3	9.92	0,0116	Derl-lilce domain family, member 3 (DERL3), variant 3, rnRNA [NM_198440]
**Nucleic acid binding and/or Response to virus** **pvalue: 2.0 × 10**^**-4**^ **FDR1.5 × 10**^**-2**^	RIG-1	3.68	0,0496	retinoic acid inducible gene I (RIG-I) [NM_014314]
	DHX58	3.23	2,44E-05	DEXH (ASp-Glu-X-His) box polypeptide 58 (DHX58),[NM_024119]
	ZBP1	3.36	0,0012	Z-DNA binding protein 1 (ZBP1), variant 1, [NM 030776]
	EIF2AK2	4.79	0,0 182	eukaryotic translation initiation factor 2-alpha kinase 2 (EIF2AK2), [NM_002759]
	PARP12	2.50	0,050	poly (ADP-ribose Polyrnerace family. member 12 (PARP12). [NM_022750]
**Other**	CMPK2	14.02	0,0036	cytidine monophosphate kinace 2. mitochondrial (CMPK2L [NM_207315]
	LAMP3	8.28	0,0128	lysosornal-acsociated membrane protem 3 (LAMP3), [NM_0 14398]
	SPATS2T	5.77	0,0080	spermatogenisis associated serine-rich 2-like (SPAT2L) [NM_001100422]
	USP4I	4.53	0,0182	partial mRNA for ubiquitin-specific protease 41 (USP41 gene). [AJ586979]
	PROM2	5.73	0,0406	prominin 2 (PROM2) variant 3, [NM_144707]
	DNHD1	5.58	0,0241	dynein heavy chain domain 1 (DNHD1), variant 2, [NM 173589]
	L0C64494	5.09	0,0249	cDNA FLJ455O9 fis, clone BRTHA2020811. [AK127417]
	IFIT5	4.86	0.0 128	interferon-induced protein with tetratricopeptide repeats 5 (IFIT5), [NM0 12420]
	LY6E	4.44	0,0101	lymphocyte antigen 6 complex. locus E çLY6E), variant I, [NM U02.s4b]
	INPP5F	3.48	0,0122	inosito1po1yphnphale-S-phnsphalaceF(fNPPSF) variant 1 [N1\it 014917]
	TTC21A	3.14	0,0462	tetratricopeptide repeat domain 21A (TTC21A), variant 2, [NM 145755]
	FGD2	2.71	0,0560	FYVE, RhoGEF and PH domain containing 2 (FDG2) [NM_173558]
**Family Q**				**Genes upregulated in asymptomatic versus severely affected individuals**
**Biological process**	**Gene**	**FC**	**Adjpvalue**	**Description**
	SMAD5	5.35	0.0140	SMAD family member 5 (NM_001001419)
	KLF15	3.58	0.0142	Kruppel-like factor 15 (006814)
	ATXN7	10.26	0.0144	ataxin 7 (NM_001128149)
	UTF1	4.59	0.0158	undifferentiated embronic cell transcription factor (NM_003577)
	ALX4	4.00	0.0179	ALX homeobox 4 (ENST00000329255)
	MAFA	3.65	0.0182	v-maf musculoaponeuroticfibrosarcoma oncogene homolog A(NM_201589)
	RARA	17.22	0.0219	retinoic acid receptor, alpha (NM_001024809)
	Tead1	5.20	0.0220	TEA domain family member 1(NM_021961)
	NEUROG3	5.51	0.0237	neurogenin 3 (NM_020999)
	DYRK1B	4.48	0.239	dual-specificity tyrosine -(Y) phosporylation regulated kinese 1B (NM_004714)
	EIE5AL1	23.96	0.2399	eukaryotic translation initiation factor 5A (NM_001099692)
**Transcription activator activity** **pvalue : 5.7 × 10-**^**4**^ **FDR: 7.1 × 10**^**-2**^	MYOG	16.54	0.0239	myogenin (mygenic factor 4 ) (NM_002479)
	GLI2	6.86	0.0258	GLI family zinc finger 28 (NM_005270)
	TFE3	3.48	0.0272	transcription factor binding to IGHM enhancer 3(NM00651)
	EVXI	7.82	0.0273	even-skipped homebox 1 (NM_005163)
	FOXC1	4.44	0.0291	forkhead box C1 (NM_001989)
	HRH1	2.91	0.0303	histamine receptor H1 (NM_001098213)
	AKT1	4.76	0.0303	v-akt murine thymoma viral oncogene homolog 1(NM_005163)
	CTRC1	7.89	0.3311	CREB regulated transcription coactivator 1 (NM_001098482)
	CARD11	7.10	0.0318	caspase recruitement domain family, member 11 (AK097139)
	CD80	3.53	0.0335	CD80 molecule RG Homo sapiens (NM_001042454)
	IL17F	4.13	0.0351	interleukin 17F (NM_021784)
	RARG	7.08	0.369	retinoic acid receptor, gamma (NM_000966)
	TGTBI111	4.38	0.0369	transforming growth factor beta
	FOXA2	2.89	0.0411	forkhead box A2 (NM_021784)
	BHLHE23	5.27	0.0420	basic helix-loop-helix- family, member e23 (NM_080606)
**Structural molecule activity** **pvalue: n.s.** **FDR: n.s.**	SPTBN2	3.36	0.0084	spectrin, beta, non-erythocytic 2 (NM_006946)
	AGRN	5.02	0.0088	agrin (NM_198576)
	CLDN19	3.82	0.0094	claudin 19 (NM_001123395)
	KRT8	3.48	0.0102	keratin 8 (NM_002273)
	COL5A1	6.53	0.0142	collagen, type V, alpha 1 (NM_000093)
	KRTAP1	3.54	0.00170	keratin associated protein 1-3 (NM_030966)
	KRT7	8.75	0.0179	keratin 7 (NM_005556)
	EMILIN1	3.04	0.0186	elastin mocrofibril interfacer 1 (NM_007046)
	COL27A1	6.30	0.0204	collagen, type XXVII, alpha 1 (NM 032888)
	MUC6	4.66	0.0271	mucin 6 (NM_005961)
	KRT1	6.10	0.0307	keratin 1 (NM_006121)
	KRT73	5.67	0.0307	lamin A/C (NM_170707)
	MUC3B	4.89	0.0311	mucin 3B, cell surface associated (ENST00000379458)
	COL11A2	8.72	0. 0369	collagen, type XI, alpha 2 (NM_001163771)
	EVPL	4.72	0.0401	evoplakin (NM_170707)
	KRT81	5.89	0.425	keratin 81 (NM_002281)
	CHAD	3.72	0.0473	chondroadherin (NM_001267)
	LAMC3	4.26	0.0473	laminin, gamma 3 (NM_006059)

Gene ontology was performed using the Database for Annotation, Visualization and Integrated Discovery (DAVID). For this analysis, biological processes associated to each transcript were used to establish significant functional categories among the top results in each family. Ontology results from the DR (2) and Q families (2) are shown.

#### Family DR

In the DR family, DR0 is a 52-year-old woman suffering from 1-3 cutaneous edema episodes per year in the absence of treatment. Her mutation-carrying relatives DR1, DR3, DR4 (three males of ages ranging from 26 to 81) have been free of symptoms for the last two years or longer. None of the three males is receiving HAE-related treatments and, what is more, DR1 has remained asymptomatic even during ACE-i treatment, which is a potent edema-precipitating drug in HAE patients [11].

**Figure 1:**
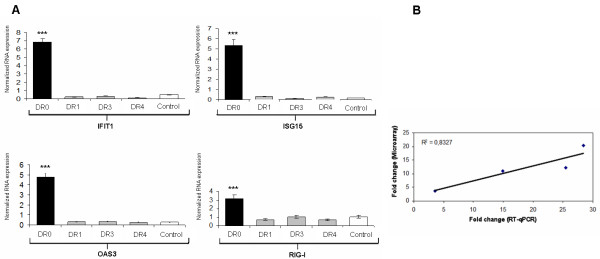
**RT-qPCR validation of the microarray results in the DR family.** Averaged results of the quantification of IFIT1, ISG15, OAS3 and RIG-I mRNAs from two independent samples are shown **A**). Correlation between RT-qPCR and microarray results **B**). The Mann-Whitney statistic was used to verify significant differences among groups. *** denotes pvalue < 0.001.

Analysis of the transcriptome in the DR family revealed significant upregulation (FC > 2; adjpvalue < 0.05) of 35 genes in DR0, 27 of which are upregulated above 4FC (Table 2). Top hits in this patient are: RSAD2, IFIT1, IFI44L, CMPK2, ISG15, HERC5, OAS3 and IFI44. Validation of the results was performed by RT-qPCR quantification of ISG15, IFIT1, OAS3 and RIG-I in DR family members (correlation coefficient R^2^:0.8327) (Figure 1). Among the remaining upregulated genes in DR0, the RNA helicase RIG-I (FC: 3,68) is relevant because it is considered a very specific signature of the presence of virus-derived RNA species into host cells [12,13]. Gene Ontology (GO) analysis confirmed enrichment of GO terms related to the biological response to virus (pvalue:7. 3 × 10^-7^; FDR:1.2 × 10^-4^), RNA binding (pvalue: 2.0 × 10^-4^; FDR:1.5 × 10^-2^) and endoplasmic reticulum-associated catabolic process (pvalue: 3.8×10^-2^; FDR:4. × 10^-2^). We did not identify transcripts significantly downregulated above the imposed threshold of -2FC in DR0 as compared to the remaining DR family members. The complete raw data are accessible as Additional file 1: Table S1, Additional file 2: Table S2, Additional file 3: Table S3).

**Figure 2:**
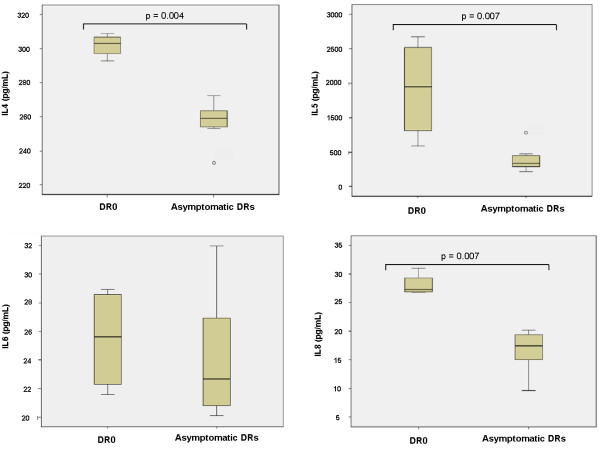
**Quantification of cytokines in plasma of the DR family.** Measurement was performed with a magnetic-bead-based multiplex immunoassay (Bio-Rad). Averaged results from two independent samples are shown. The medium bar in the box and whisker plots corresponds to the median from two independent samples. In both assays, plasma from patient DR0 had significantly higher concentrations of IL4, IL5 and IL8 than control plasmas and asymptomatic patients. IL6 was also higher in DR0, although it did not reached significance. Statistical analyses were performed with the Mann-Whitney test.

We also measured cytokines’ levels in the sera of DR family members in two independent samples obtained in a 10 months interval. In both measurements, DR0 exhibited significantly higher expression of IL4, IL5 and IL8 as compared to healthy controls and symptoms-free relatives (with or without mutation in the *C1INH* locus) (Figure 2). This cytokine expression pattern suggests a Th2 response and, therefore, the involvement of humoral immunity. IL4 stimulates B cells and induces immunoglobulin class switching to IgE. It also has a role in chronic inflammation and wound repair by modulating the activity of macrophage subpopulations. IL5 is produced by Th2 and mast cells. It is involved in B cell growth stimulation, immunoglobulin production and activation of eosinophils. As such, its overproduction has been associated to allergic disease and asthma [14]. IL8 is a key mediator of inflammation. It is produced mainly by macrophages, endothelial and epithelial cells, although it can be secreted by a variety of cells upon TLR stimulation. It promotes chemotaxis in neutrophils and maintains proinflammatory conditions by inducing further increase in oxidant stress mediators, thus being implicated in chronic inflammatory diseases [15].

Whether or not this cytokine profile is directly related to the gene expression pattern observed in the microarray of the patient is beyond the score of this paper. But taking under consideration the outcome of the microarray in DR0, the fact that similar results were obtained in the quantification of serum cytokines in two time-elapsed samples, and the absence of infectious manifestations in the patient, the picture is suggestive of a chronic, asymptomatic process. By analyzing the patient’s serum antibodies, we discarded a putative Influenza infection as causative of the gene expression pattern observed in DR0 (data not shown).

All considered, the rationale underlying these results may be that, in the DR family, long-lasting, chronic viral infections are a major triggering factor of angioedema. Furthermore, it is feasible that genetic variation in one or several genes involved in the immune response against viruses and immune system regulation could influence both the course of infection and the development of HAE symptoms.

On the other hand, the three asymptomatic males in the DR family exhibit upregulation of 32 transcripts related to diverse biological functions that do not constitute significant functional clusters, but share functional links among them. That is the case of OLIG1 and OLIG2 transcription factors, which are expressed mainly during central nervous system development [16], and RAVER and PTBP1, both implicated in binding to polypyrimidine tract sequences and regulating the expression of alternative splicing forms [17].

#### Family Q

Two members of this family carrying the R472X substitution in C1-INH (Q3 and Q10) are asymptomatic and do not require specific treatment for HAE. Examination of the transcriptome in the asymptomatic individuals in this family reveals relative upregulation of 394 transcripts whose clustering, according to the transcripts’ associated biological processes, renders only one significant group of 29 genes with “Transcriptional activation activity” (pvalue: 5.7 × 10^-4^; FDR: 7.1 × 10^-2^) (Table 2). Within this set of genes, IL17F is upregulated above 4fold, which could be related to the induction of proinflammatory responses; however, it could not be measured in the patient’s plasma.

Another consistent set of genes upregulated in asymptomatic mutation carriers from this family which is associated to “Structural molecule activity” is composed of 23 transcripts (including those of 6 keratin genes, 4 collagen genes and 2 laminin genes) although pvalue and FDR score of this cluster do not reach statistical significance.

On the other hand, when compared either with asymptomatic or control individuals, severely affected members of the Q family exhibit an expression pattern characterized by the upregulation of 43 genes which take part in diverse biological processes. Nevertheless, no significant functional cluster could be detected.

#### Family AR

Most of the members carrying the R472X substitution in this family (AR0, AR2, AR3, AR4, AR5, AR7, AR8, AR9) suffer frequent and mild-to-severe abdominal and/or cutaneous symptoms of HAE. Among them, AR2, AR3 and AR4 are under treatment with attenuated androgens (Danazol) while their remaining relatives, who experience milder symptoms, do not receive any prophylactic treatment. Only one member of this family, AR10, a 53-year old man, has never manifested clinical signs of HAE nor has been treated for the disease and his diagnosis was achieved only when his affected daughter was studied. No significant disease-modifying factors could be identified in this family.

## Discussion

The aim of the present study was to investigate the influence of the PBMC transcriptome on the predisposition to or protection from developing HAE symptoms. The results presented shed more light on the interface between innate and adaptive immunity in the context of C1-INH deficiency and question the role of local C1-INH synthesis by PBMCs in HAE patients. Effectively, not the PBMCs synthesis of C1-INH mRNA, nor that of other molecules classically implicated in the pathogenesis of HAE like Angiotensin Converting Enzyme (ACE), Bradykinin Receptors 1 and 2, (BDKR1, BDKR2) or Aminopeptidase P (APP) showed any statistical effect on the frequency of HAE attacks [18,19]. In agreement with that, instead of a common disease-associated signature, PBMCs from the three families analyzed exhibit different expression patterns linked to their symptoms. What is more, the most significant results in the present work were obtained in the symptomatic individual from the DR family, whose expression profile strongly suggests host response to viral infection as the major disease-precipitating factor. This facts might indicate that local protein synthesis by the PBMC milieu plays only a modest role under the circumstances analyzed here (in a free-of-symptoms period).

This interpretation of the results differs from reports by Klausegger et al. describing a child affected of Evans syndrome and HAE, whose angioedema symptoms were clinically and biochemically cured by stem cell bone marrow transplantation [20]. Although the authors report a complete cure of HAE signs which is supported by the rising in serum C1-Inh function from 5-50% to 84% of control values, circulating levels of C4 in the patient are still at the lower limit of normality two years after transplantation (11.2 mg/dL). Besides, he is still in his childhood years, and given that HAE tends to manifest or worsen at puberty, a longer follow-up period would be recommendable to confidently assess full recovery of normal C1-Inh synthesis. On the other hand, the apparent discrepancy between the case reported by Klausegger et al. and the results presented here could in fact reflect a lack of statistical power in our experimental design, either by the reduced sample size or due to the small effect of the investigated disease-modifiers in the PBMCs. In that case, a more robust design of the study (e.g. RNA analysis of patients in the course of an attack *versus* asymptomatic patients) might overcome the statistical difficulties. Alternatively, the interrogation of a different tissue -like biopsy derived endothelial cell or hepatocyte cultures- could be a more appropriate experimental setting, although the difficulty of obtaining an adequate sample size from patients’ biopsies is evident. In the case of the present study, samples from different tissues were initially considered, but PBMCs were chosen as the most affordable option.

Regarding the results obtained in the DR family, we measured RNA expression of upregulated genes ISG15, IFIT1, OAS3 and RIG-I by RT-qPCR with TaqMan primers and probes. In all cases, the results of microarray and RT-qPCR experiments correlated adequately, for an overall correlation coefficient of R^2^:0.8327 (Figure 1). We did not validate the microarray results at the protein level due to the lack of a convenient commercial kit, but we did intend to characterize the cytokine profile in the sera from the DR family members with a magnetic-bead-based multiplex immunoassay. The results from two independent samples demonstrated higher serum levels of IL4 (pvalue:0.004), IL5 (pvalue:0.007) and IL8 (pvalue:0.007) in the symptomatic patient DR0 as compared to both healthy controls and asymptomatic DRs. Given that the two samples analyzed were obtained within a 10 months interval and based on the microarray results, this cytokine profile could reflect chronic inflammation or a long-lasting, asymptomatic infection in the patient.

The role for adaptive immunity in the development of HAE has remained controversial along the years. HAE symptomatology is accompanied by a higher prevalence of autoimmunity including glomerulonephritis, lupus-like disorders and Sjögren’s syndrome [21]. In the context of a C1-INH deficiency, abnormal activation of complement via the classical pathway consumes the initial components of the cascade and may expose HAE patients to an increased risk of immune-complex disease, as seen in the C2 or C4 deficiencies.

Several reports exist that describe immunoregulatory alterations in HAE ranging from autoimmunity (glomerulonephritis and Sjögren’s syndrome being the most frequent conditions) to unbalanced T cell subpopulations or polyclonal B cell activation [22]. However, while some recent publications in the field found a higher prevalence of autoimmune disorders but failed to detect further immunoregulatory defects [23], others have shown abnormalities in the biology of HAE memory B cells that provide a rationale for the high prevalence of autoimmunity observed in this disease [24]. The latter include higher overall phosphotyrosine levels and enhanced expression of CD69, CD5, CD21 and Toll-Like Receptor 9 (TLR-9) in memory B cells of HAE patients as compared to controls. What is more, Kessel and colleagues demonstrated that TLR-9 expression was higher in the memory B cells of those HAE cases where autoantibodies were also present, suggesting that the enhanced production of autoantibodies in this disorder is most probably due to increased B cell activation.

The implication and importance of adaptive immunity in the development of HAE is also supported by clinical observations in series of patients. Cedzyński and colleagues analyzed the effect of infectious disease on the clinical scores for the frequency and severity of HAE symptoms. Their results, although not reaching significance, showed that infection by *Helicobacter pylori* and *Hepatitis B virus* may slightly worsen HAE pathology [25]. Infection by *H. pylori* has also been reported to cause acquired C1-INH deficiency in a case report by Farkas and colleagues. The authors attributed this effect to the excessive consumption of complement by antibodies directed against *H. pylori*. Moreover, successful eradication of the pathogen in the patient resulted in a fivefold reduction in- or complete disappearance of HAE symptoms [26]. Thus, it is predictable that infectious diseases resulting in enhanced antibody response and the formation of immune complexes may trigger the overuse of already reduced C1-INH in HAE patients [27].

In contrast to the DR family, symptomatic members from the Q family show no evidence of viral infection as a noteworthy precipitating factor. Instead, the transcriptome signature observed in these individuals is more complex and consists of 43 genes upregulated in the severely affected Q members *versus* 394 transcripts showing higher expression in their symptom-free relatives. Given the high number of genes with altered expression in this family and their diverse clustering options, it is difficult to give a simple interpretation to the results. As shown in Table 2, gene ontology analysis renders only one significant cluster of genes associated to transcription activator activity.

Regarding the AR family, where no significant hit was detected, it is likely that the high proportion of ARs receiving androgen prophylactic treatment at the time of sampling (5 out of 8 patients) would wipe away the individual differences of gene expression and explain the lack of overall results in the family.

The present results arise from the study of a very reduced number of HAE families harbouring the same HAE type I mutation. It is likely that part of the expression profiles characterized in this cohort represent only patient- and/or mutation-specific signatures, rather than general mechanisms of the disease.

All considered, our data did not provide new major evidences concerning the role of PBMCs in the development of HAE in basal, out of crises, situations. The strong association between asymptomatic viral infection and HAE severity observed in the DR family supports the idea of infectious diseases as a major precipitating factor, which is endorsed by abundant clinical observations [6,24]. However, data from a single family cannot be extrapolated further and large scale, systematic screening studies would be needed to statistically associate the mRNA expression pattern observed in the DR0 patient with predisposition to HAE flares.

## Abbreviations

HAE: Hereditary angioedema; C1-INH: C1 Inhibitor protein; C1INH: C1 Inhibitor gene; FXII: Coagulation factor XII; PBMCs: Peripheral blood mononuclear cells; FC: Fold change; FDR: False discovery rate.

## Competing interest

The authors declare that they have no competing interests.

## Authors’ contributions

ALL analyzed microarray data, performed the validation of the results and wrote the manuscript. FSC carried out hybridization and microarray procedures. SG took prepared the biological samples and took part in the validation of the results. AD participated in the experimental design and provided valuable advice for the analyses of the results. MLT planned the study and took part in the interpretation of the results and writing of the manuscript. All authors read and approved the manuscript.

## Supplementary Material

Additional file 1: Table S1 DR family. Genes upregulated in Asymptomatic versus Control individuals. Click here for file

Additional file 2: Table S2 DR family. Genes upregulated Symptomatic versus Control individuals. Click here for file

Additional file 3: Table S3 Q family. Genes upregulated in Asymptomatic versus Control individuals. Click here for file

## References

[B1] Caballero T Baeza ML Cabañas R Campos A Cimbollek S Gómez-Traseira C González-Quevedo T Guilarte M Jurado-Palomo J Larco JI López-Serrano MC López-Trascasa M Marcos C Muñoz-Caro JM Pedrosa M Prior N Rubio M Sala-Cunill A Spanish Study Group on Bradykinin-Induced Angioedema (SGBA) Consensus statement on the diagnosis, management, and treatment of angioedema mediated by bradykinin. Part I. Classification, epidemiology, pathophysiology, genetics, clinical symptoms, and diagnosis J Investig Allergol Clin Immunol 2011 21 5 333 347 21905496

[B2] Cichon S Martin L Hennies HC Müller F Van Driessche K Karpushova A Stevens W Colombo R Renné T Drouet C Bork K Nöthen MM Increased activity of coagulation factor XII (Hageman factor) causes hereditary angioedema type III Am J Hum Genet 2006 79 6 1098 1104 10.1086/509899 17186468 PMC1698720

[B3] Bossi F Peerschke EI Ghebrehiwet B Tedesco F Cross-talk between the complement and the kinin system in vascular permeability Immunol Lett 2011 140 1–2 7 13 21762728 10.1016/j.imlet.2011.06.006 PMC3162365

[B4] Joseph K Tuscano TB Kaplan AP Studies of the mechanisms of bradykinin generation in hereditary angioedema plasma Ann Allergy Asthma Immunol 2008 101 3 279 286 10.1016/S1081-1206(10)60493-0 18814451

[B5] Nussberger J Cugno M Amstutz C Cicardi M Pellacani A Agostoni A Plasma bradykinin in angio-oedema Lancet 1998 351 9117 1693 1697 10.1016/S0140-6736(97)09137-X 9734886

[B6] Agostoni A Aygören-Pürsün E Binkley KE Blanch A Bork K Bouillet L Bucher C Castaldo AJ Cicardi M Davis AE De Carolis C Drouet C Duponchel C Farkas H Fáy K Fekete B Fischer B Fontana L Füst G Giacomelli R Gröner A Hack CE Harmat G Jakenfelds J Juers M Kalmár L Kaposi PN Karádi I Kitzinger A Kollár T Kreuz W Lakatos P Longhurst HJ Lopez-Trascasa M Martinez-Saguer I Monnier N Nagy I Németh E Nielsen EW Nuijens JH O’grady C Pappalardo E Penna V Perricone C Perricone R Rauch U Roche O Rusicke E Späth PJ Szendei G Takács E Tordai A Truedsson L Varga L Visy B Williams K Zanichelli A Zingale L Hereditary and acquired angioedema: problems and progress: proceedings of the third C1 esterase inhibitor deficiency workshop and beyond J Allergy Clin Immunol 2004 114 3 Suppl S51 S131 15356535 10.1016/j.jaci.2004.06.047 PMC7119155

[B7] Cillari E Misiano G Aricò M La Rocca E Lio D di Leonardo S Brai M Modification of peripheral blood T-lymphocyte surface receptors and Langerhans cell numbers in hereditary angioedema Am J Clin Pathol 1986 85 3 305 311 2944374 10.1093/ajcp/85.3.305

[B8] Brickman CM Tsokos GC Chused TM Balow JE Lawley TJ Santaella M Hammer CH Linton GF Frank MM Immunoregulatory disorders associated with hereditary angioedema. II. Serologic and cellular abnormalities J Allergy Clin Immunol 1986 77 5 758 767 10.1016/0091-6749(86)90425-2 3486201

[B9] Gelfand JA Sherins RJ Alling DW Frank MM Treatment of hereditary angioedema with danazol. Reversal of clinical and biochemical abnormalities N Engl J Med 1976 295 26 1444 1448 10.1056/NEJM197612232952602 792688

[B10] Bolstad BM Irizarry RA Astrand M Speed TP A comparison of normalization methods for high density oligonucleotide array data based on variance and bias Bioinformatics 2003 19 2 185 193 10.1093/bioinformatics/19.2.185 12538238

[B11] Vasekar M Craig TJ ACE inhibitor-induced angioedema Curr Allergy Asthma Rep 2012 12 1 72 78 10.1007/s11882-011-0238-z 22127615

[B12] Kato H Takeuchi O Sato S Yoneyama M Yamamoto M Matsui K Uematsu S Jung A Kawai T Ishii KJ Yamaguchi O Otsu K Tsujimura T Koh CS Reise Sousa C Matsuura Y Fujita T Akira S Differential roles of MDA5 and RIG-I helicases in the recognition of RNA viruses Nature 2006 441 7089 101 105 10.1038/nature04734 16625202

[B13] Co JG Witwer KW Gama L Zink MC Clements JE Induction of innate immune responses by SIV in vivo and in vitro: differential expression and function of RIG-I and MDA5 J Infect Dis 2011 204 7 1104 1114 10.1093/infdis/jir469 21881126 PMC3164431

[B14] Greenfeder S Umland SP Cuss FM Chapman RW Egan RW Th2 cytokines and asthma. The role of interleukin-5 in allergic eosinophilic disease Respir Res 2001 2 2 71 79 Review 10.1186/rr41 11686868 PMC59571

[B15] Mukaida N Pathophysiological roles of interleukin-8/CXCL8 in pulmonary diseases Am J Physiol Lung Cell Mol Physiol 2003 284 4 L566 L577 Review 12618418 10.1152/ajplung.00233.2002

[B16] Li H Richardson WD The evolution of Olig genes and their roles in myelination Neuron Glia Biol 2008 4 2 129 135 10.1017/S1740925X09990251 19737433 PMC6326352

[B17] Spellman R Rideau A Matlin A Gooding C Robinson F McGlincy N Grellscheid SN Southby J Wollerton M Smith CW Regulation of alternative splicing by PTB and associated factors Biochem Soc Trans 2005 33 Pt 3 457 460 15916540 10.1042/BST0330457

[B18] Freiberger T Grombiříková H Ravčuková B Jarkovský J Kuklínek P Kryštůfková O Hanzlíková J Daňková E Kopecký O Zachová R Lahodná M Vašáková M Grodecká L Litzman J No evidence for linkage between the hereditary angiooedema clinical phenotype and the BDKR1, BDKR2, ACE or MBL2 gene Scand J Immunol 2011 74 1 100 106 10.1111/j.1365-3083.2011.02547.x 21375555

[B19] Duan QL Nikpoor B Dube MP Molinaro G Meijer IA Dion P Rochefort D Saint-Onge J Flury L Brown NJ Gainer JV Rouleau JL Agostoni A Cugno M Simon P Clavel P Potier J Wehbe B Benarbia S Marc-Aurele J Chanard J Foroud T Adam A Rouleau GA A variant in XPNPEP2 is associated with angioedema induced by angiotensin I-converting enzyme inhibitors Am J Hum Genet 2005 77 4 617 626 10.1086/496899 16175507 PMC1275610

[B20] Klausegger A Wiednig M Urban C Lackner H Reiter H Bauer JW Aberer W Successful allogeneic cord blood transplantation in a patient with Evans syndrome leads to correction of hereditary angioedema type I as secondary effect Bone Marrow Transplant 2012 47 9 1259 1261 10.1038/bmt.2012.7 22327128

[B21] Brickman CM Tsokos GC Balow JE Lawley TJ Santaella M Hammer CH Frank MM Immunoregulatory disorders associated with hereditary angioedema. I. Clinical manifestations of autoimmune disease J Allergy Clin Immunol 1986 77 5 749 757 10.1016/0091-6749(86)90424-0 3084606

[B22] Farkas H Csuka D Gács J Czaller I Zotter Z Füst G Varga L Gergely P Lack of increased prevalence of immunoregulatory disorders in hereditary angioedema due to C1-inhibitor deficiency Clin Immunol 2011 141 1 58 66 Epub 2011 10.1016/j.clim.2011.05.004 21636327

[B23] Kessel A Peri R Perricone R Guarino MD Vadasz Z Novak R Haj T Kivity S Toubi E The autoreactivity of B cells in hereditary angioedema due to C1 inhibitor deficiency Clin Exp Immunol 2012 167 3 422 428 10.1111/j.1365-2249.2011.04527.x 22288585 PMC3374274

[B24] Cedzyński M Madaliński K Gregorek H Swierzko AS Nowicka E Obtułowicz K Dzierzanowska-Fangrat K Wojda U Rabczenko D Kawakami M Possible disease-modifying factors: the mannan-binding lectin pathway and infections in hereditary angioedema of children and adults Arch Immunol Ther Exp (Warsz) 2008 56 1 69 75 10.1007/s00005-008-0004-7 18250972 PMC2734250

[B25] Farkas H Gyeney L Majthényi P Füst G Varga L Angioedema due to acquired C1-esterase inhibitor deficiency in a patient with Helicobacter pylori infection Z Gastroenterol 1999 37 6 513 518 10427658

[B26] Farkas H Füst G Fekete B Karádi I Varga L Eradication of Helicobacter pylori and improvement of hereditary angioneurotic oedema Lancet 2001 358 9294 1695 1696 10.1016/S0140-6736(01)06720-4 11728547

[B27] Frank MM Gelfand JA Atkinson JP Hereditary angioedema: the clinical syndrome and its management Ann Intern Med 1976 84 5 580 593 10.7326/0003-4819-84-5-580 1275365

